# IMU Consensus Exception Detection with Dynamic Time Warping—A Comparative Approach

**DOI:** 10.3390/s19102237

**Published:** 2019-05-14

**Authors:** Chan-Yun Yang, Pei-Yu Chen, Te-Jen Wen, Gene Eu Jan

**Affiliations:** 1 Department of Electrical Engineering, National Taipei University, New Taipei City 23741, Taiwan; s305713@gmail.com; 2 Department of Computer and Communication Engineering, Taipei City University of Science & Technology, Taipei 11202, Taiwan; tzwen@tpcu.edu.tw; 3 Graduate Institute of Animation and Film Art, Tainan National University of the Arts, Tainan City 72045, Taiwan

**Keywords:** dynamic time warping, remote abnormality detection, the inertial measurement unit

## Abstract

A dynamic time warping (DTW) algorithm has been suggested for the purpose of devising a motion-sensitive microelectronic system for the realization of remote motion abnormality detection. In combination with an inertial measurement unit (IMU), the algorithm is potentially applicable for remotely monitoring patients who are at risk of certain exceptional motions. The fixed interval signal sampling mechanism has normally been adopted when devising motion detection systems; however, dynamically capturing the particular motion patterns from the IMU motion sensor can be difficult. To this end, the DTW algorithm, as a kind of nonlinear pattern-matching approach, is able to optimally align motion signal sequences tending towards time-varying or speed-varying expressions, which is especially suitable to capturing exceptional motions. Thus, this paper evaluated this kind of abnormality detection using the proposed DTW algorithm on the basis of its theoretical fundamentals to significantly enhance the viability of the methodology. To validate the methodological viability, an artificial neural network (ANN) framework was intentionally introduced for performance comparison. By incorporating two types of designated preprocessors, i.e., a DFT interpolation preprocessor and a convolutional preprocessor, to equalize the unequal lengths of the matching sequences, two kinds of ANN frameworks were enumerated to compare the potential applicability. The comparison eventually confirmed that the direct template-matching DTW is excellent in practical application for the detection of time-varying or speed-varying abnormality, and reliably captures the consensus exceptions.

## 1. Introduction

The technique of pattern recognition has been adopted in a wide variety of applications, such as speech recognition in engineering, traffic analysis and control in civil administration, stock exchange forecasting in economics, and classification of rocks in geology [1]. According to the original idea of illuminating and inspiring the evolution of pattern recognition technology, the approaches can be roughly divided into four main groups, as follows: template matching, syntactic and structural bifurcation detection, statistical learning approach, and neural network inference [2]. The process of template matching involves recognizing an undetermined pattern from a predefined template. It is often achieved by comparing the similarity between the candidate pattern and predefined patterns. The metric of the similarity measurement can be defined by the correlation between the candidate pattern and a certain predefined template [3]. Under ordinary conditions, a higher correlation metric implies greater similarity between the candidate pattern and the predefined pattern, and thus the highest one is normally recommended as the matched pattern. The matching procedure could even be manipulated in the patterns’ original feature domain or a transformed feature domain. Generally, the matching skill relies on the performance by which the features of the domain can be maintained. Of course, patterns matched in their original format without any transformation should be the easiest and most direct method in this case, as this does not lose too much recognition capability. For example, the time domain and spatial domain are often priority selections for acoustic recognition and image recognition, respectively, in their applications. In the original feature domain, the matching method can be manipulated rapidly straightforwardly. This kind of direct pattern matching is also advantageous for identifying and extracting candidate consensus templates from a continuous pattern flow. In continuous template matching applications, consensus templates are often captured and stored in advance in a library, without complicated format transformation. Based on the stored format, the template could be relatively easily subjected to straightforward matching manipulations in the continuous signal flow. Also, the direct storage of the templates is beneficial to the maintenance and updating of their corresponding libraries, on which the continuous mechanism greatly relies.

There are many template matching applications for machine vision [4]. Hu et al. proposed a method for detecting and tracking ground objects from flying vehicles [5]. Brown surveyed currently existing image registration techniques based on template matching [6]. Popular high-level applications include the navigation of mobile robots, camera self-calibration, and 3D reconstruction by speeded-up robust features (SURF) [7]. Acoustic recognition is also a category of the template matching application [8]. By recording spoken words and phrases as speech templates for acoustic recognition, there are many recognition techniques that can then be used for identifying this kind of template in continuous speech. In conventional techniques for acoustic recognition, applications are subdivided into five sub-categories as follows: template-based approaches, dynamic time warping, knowledge-based approaches, artificial intelligence approaches, and statistical approaches [9]. Among these categories, dynamic time warping (DTW) is advantageous due to its merits in overcoming the issue of time/speed synchronization for patterns in continuous flow [10]. In 2009 [11], Fang showed that both DTW and HMM (Hidden Markov Model [12]) are intrinsically nonlinear sequence synchronized algorithms, and that they share the same core idea of dynamic programming. The auto-synchronization characteristic makes their algorithmic varieties more flexible and practical for template matching applications, and one such application involves matching temporal sequences continuously in their corresponding feature domain. Although DTW has been widely known in the field of acoustic recognition for a long time, it has also been proposed for the purposes of functional verification and authentication tasks in the last decade. For example, in 2010, Derawi et al. reported it with wearable sensors to develop a stable cycle detection mechanism for improved biometric gait recognition [13]. Since then, it has been utilized in Kinect applications for robust hand gesture recognition [14] and gesture-based user identification and authentication [15] by Wu et al. and Raheja et al., respectively. 

In this study, an inertial measurement unit (IMU) was utilized to record human movement and motion. In this kind of application, the acquired signals might vary with motion time or speed, and thus they were unsynchronized. DTW deserves particular attention with respect to identifying a specific motion pattern from a nonlinearly continuous and unsynchronized sequence flow. Due to its small size and wireless communication, IMU, which is often a wearable device, is appropriate for collecting human motion data comfortably and with less inconvenience. The advantages of the wearable IMU make it feasible for applications such as measuring energy expenditure and evaluating the performance of sports for healthcare. The first work combining DTW and IMU devices appeared in 2006 [16]. The programming-by-example gesture recognition work introduced by Gabayan and Lansel was just a preliminary report with only fragmentary and rough descriptions, and barely available references. The combination of IMU devices and DTW was employed again to develop hand gesture recognition by Barczewska and Drozd in 2013 [17]. This category of hand tracking gesture registration emphasizes stability, with the gesture being able to be robustly or even uniquely identified.

The study extended the application of IMU devices to a sort of on-line monitoring and detection system, especially with respect to the potential application in cases that are subject to abnormal exceptions. In contrast to the robustness required by the applications of hand gesture registration, this kind of application places greater emphasis on the sensitivity to the exceptions, which is indeed crucial to rescuing the greatest number of possible cases in time. As is widely recognized, the consensus abnormality of a common patient might occur in a way that exhibits a nonlinear, unsynchronized, random or even chaotic tendency. Despite DTW having auto-synchronization capability, a recognition methodology to capture the exceptions sensitively and to identify the consensus abnormalities in time would be more crucial when developing remote healthcare. A proper system designed using the DTW technique would practically facilitate the expectation of remote healthcare. A wearable IMU activity detection system was thus developed by our laboratory, as presented in [18], and applied in the context of post mini-stroke healthcare [19]. The present paper enhances and refines our previous developments to improve accuracy and to extend the proposed method to the real-time application of consensus abnormality detection.

## 2. Technical Primitives

In this paper, the DTW technique was adopted to detect motion activity. In order to carry out a fair comparison with the proposed methodology, the paper also introduces machine learning-based artificial neural network (ANN) inference as a contrast model. To satisfy the contrast model, the input sequences of the neural network should be equal in length; however, the motion speed of the activity might be unsteady in time, causing the length of the sequence to vary with time. Therefore, preprocessors were introduced in order to equalize the length of the inputs. Two strategies were applied here as potential candidates for the equalization, including a discrete Fourier transform (DFT) interpolation preprocessor and a convolutional preprocessor, with the aim of completely organizing the neural network pattern recognition.

### 2.1. Dynamic Time Warping

By definition, DTW [10] is sensitive to and responsible for measuring two sequences with different lengths using discrete dynamic programming. The two discrete warped sequences **x** and **z** can first be checked using different sequence sizes for *N* and *M*, respectively, i.e.,

(1) $$x\;=\;\left[x_{1}{,\;x}_{2}{,\;\ldots ,\;x}_{N}\right],\;\mathrm{and}\;z\;=\;\left[z_{1}{,\;z}_{2}{,\;\ldots ,\;z}_{M}\right]$$

**Figure 1:**
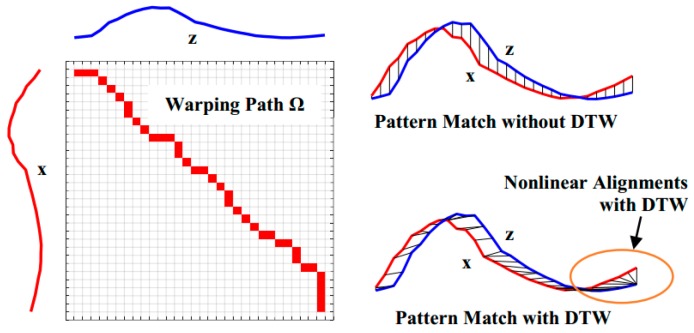
Dynamic time warping.

To align both sequences, an *N* × *M* matrix is established, as shown in Figure 1. Every element of the matrix is distance measured between sampling points *x_n_*, *n* = 1, 2, …, *N*, and *z_m_*, *m* = 1, 2, …, *M*, and denoted as a cost function *δ*(*n*, *m*). *δ*(*n*, *m*) is thus defined as

(2) $${\delta (n,\;m)\;=\;(x}_{n\;}{-\;z}_{m}{)}^{2}$$

In a recursive fashion, the DTW algorithm calculates and accumulates the cost function *δ*(*n*, *m*) throughout the whole matrix, using a minimization principle in order to accumulate the cost of two-dimensional sequentially adjacent elements, i.e.,

(3) σ(n, m) = δ(n, m) + min(σ(n − 1, m), σ(n, m − 1), σ(n − 1, m − 1))

where *σ*(*n*, *m*) are be the accumulated minimum of the fragmental distances *δ*’s from (*n*, *m*) = (1, 1) to (*n*, *m*) = (*N*, *M*) to traverse the two-dimensional matrix. According to the accumulation Rule (3), the time minimal cumulative distance *σ*(*N*, *M*) can be reached to achieve a shortened path Ω for measuring the distance between the sequences **x** and **z**. The contiguous path Ω, recorded and warped from the first cell (1, 1) to the final cell (*N*, *M*), provides the total warping cost Σ as:

(4) $$\Sigma \;=\;\sqrt{\underset{i\in \Omega }{\sum }\delta_{i}\left(n,\;m\right)}$$

The optimization rule described in (3) is a key to achieving a nonlinear auto-correlated alignment of the sequences. It is easy to verify that after the optimized correlation alignment, the score Σ is the maximum similarity between the sequences **x** and **z**, as shown in Figure 1. Lower scores of Σ imply higher similarity between sequences of unequal length. According to this alignment, the optimal warping path Ω becomes the best fit of the two sequences, and paves the way to achieving the greatest similarity of the aligned sequences, even if they are unequal in length. Here, three points are attributed to DTW to enhance the dynamic matching as follows:

- Controlled endpoint matching
- Floating adjustable local matching
- The most correlated global matching

For paired sequences of lengths *M* and *N*, the computational complexity of the DTW algorithm is *O*(*MN*) since a *M* × *N* matrix implicitly has to be built in order to recursively run the warping path Ω.

### 2.2. Neural Network Matching

**Figure 2:**
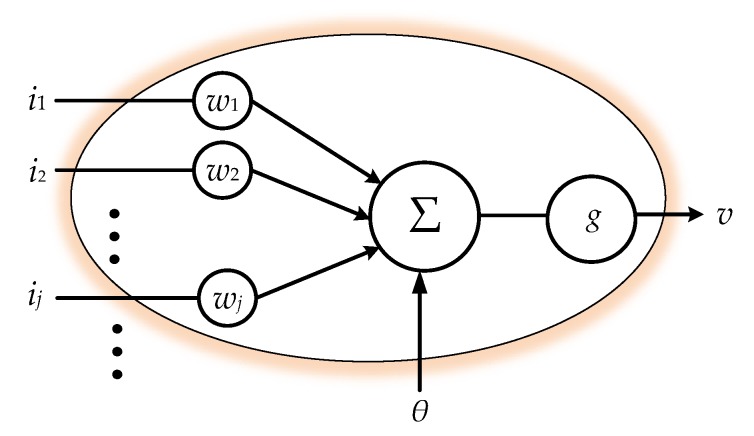
Model of a single neuron.

Machine learning techniques have been widely used to solve pattern recognition problems [20], and many related methodologies have been invented in recent decades [21]. Among these methodologies, ANN seems to be one of the most popular methods for such kinds of recognition work [22]. As a typical data-driven approach, which establishes the pattern recognition model by converging the similarity among the input features, ANN requires fundamentally homogeneous—i.e., equal length—inputs in their features’ dimensions for evolution. With input features of varying lengths, ANN is not able to proceed in calculating the similarity. Hence, we cascade a preprocessor for sequence equalization ahead of a general ANN scheme in order to achieve learning. The equalization preprocessors will be introduced in the following two sections. Neural networks conceptually imitate biological neural systems. The organization of the artificial model of the network aims to mimic the learning and function of a biological brain. The architecture of a single artificial neuron, as shown in Figure 2, can be regarded as a nonlinear activation function which gathers the inputs *i_j_*, *j* = 1, 2, …, for conventional activation:

(5) $$v=g\left(\sum_{j}w_{j}i_{j}-\theta \right)$$

where *i_j_*, *j* = 1, 2, …, are the inputs of the neuron, *w_j_* is the corresponding weight of *i_j_*, and *θ* is a real bias constant. While the linear combination of the inputs simply manipulate the signals for weighted synthesis, the activation function *g*(∙), which is inspired by biological brains, mimics a biological synaptic excitation and inhibition, transforming the activation level of the neuron into two basic state outputs: either on or off.

**Figure 3:**
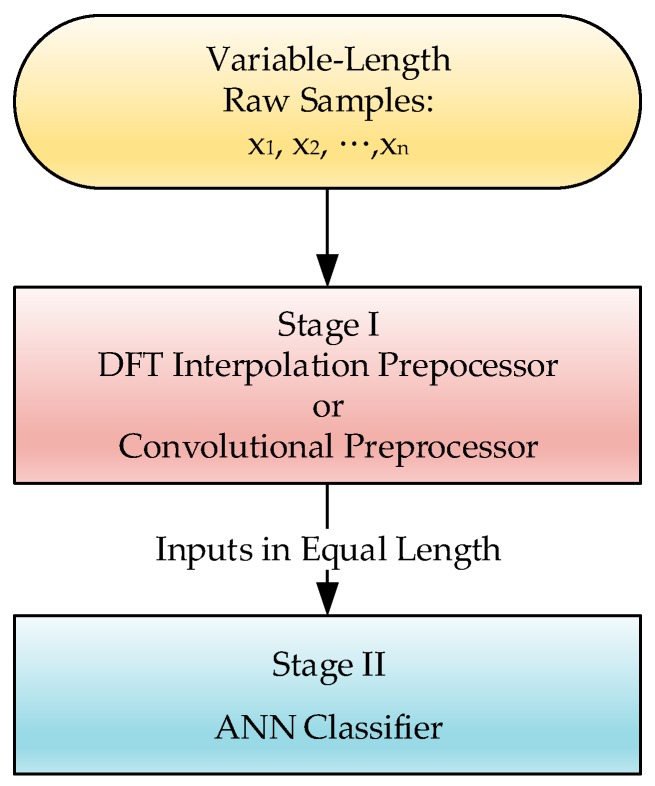
A two-stage scheme to complete the ANN model for evaluation.

In this study, the IMU motion perception employed a back-propagation neural network (BPNN), as shown in Figure 3 [22], for the experiments. Other than a forward procedure to result in the output *y*, BPNN includes an additional backward procedure to feed back an approximated convergence error. With forward and backward evolution, BPNN aggressively exhibits a capacity for accuracy in predictions. The BPNN is moved by the approximated feedback error, which can be propagated backward iteratively to determine the interconnection weights by achieving optimization of *y*. In this scheme, a delta rule, which is the gradient of an error function that is negatively proportional to the corresponding interconnection weight, is often employed to iteratively evaluate and update the interconnection weight of BPNN:

(6) $${\Delta w}_{i}=-\eta \frac{\partial E_{d}}{\partial w_{i}}$$

where Δ*w_i_* = *w_i_*_, new_ − *w_i_*_, old_, *E_d_* is the error function, and *η* is the learning ratio, 0 < *η* < 1. The approximated error would converge iteratively under a cyclic feed-forward back-propagation evolution procedure to reach an accurate prediction. The layered BPNN will well adapt the system response based on learning from actual instances. Through iteratively propagating the errors backward to update the structural interconnection weights internally, a satisfactory precision can still be obtained, even for a complex problem. 

### 2.3. Equalization Preprocessor Ahead of ANN

Even given its powerful prediction capability, ANN’s decreasing error gradient approaches its learning goal based only on the simple criterion of similarity comparison, and the comparison corresponds directly to the homogeneity of the input features. Ensuring identical input feature dimensions in order to measure similarity became, in turn, a big issue for this study, i.e., the learning machine becomes unfeasible if its corresponding inputs are of inconsistent lengths. Unfortunately, the input sequences for matching in this study would vary in length due to variations in motion speed or motion time. Therefore, we added an equalization preprocessor ahead of the ANN to make the input features consistent, and make the approach plausible. Figure 3 shows the scheme of the combinational procedure for training the ANN. Two kinds of preprocessors are employed in this study, one is a DFT interpolation preprocessor, and the other is a convolutional preprocessor.

#### 2.3.1. The DFT Interpolation Preprocessor

A DFT interpolation preprocessor has been adopted, situated in front of the model in order to make the input patterns of the ANN classifier have the same length. By using discrete Fourier transform with zero padding in the frequency domain, the length equalization of the patterns can be achieved [23]. The DFT interpolation technique was originally implemented to improve the signal resolution by means of zero padding in the frequency domain, with the resolution of the sampled signal being adjustable based on the number of padding zeros. The greater the number of padding zeros adopted, the higher the interpolation resolution of the sampled discrete signal. The DFT smooth interpolation preprocessor not only naturally increases the signal resolution, but also suppresses signal distortion. The work therefore employed DFT interpolation as a preprocessor to resolve the problem of uneven sequence length presented by the unsynchronized IMU motion data acquisition.

According to the Whittaker-Shannon interpolation theorem [24], the periodical interpolation sequence of a real number sequence in the time domain can be recovered through zero-padding processing in the corresponding frequency domain. For a given signal of finite *N* samples, denoted as **x** and **x** = [*x*_1_, *x*_2_, …, *x_n_*, …, *x_N_*]*^T^*, the DFT of **x** is

(7) $$\varphi =\left\{\varphi_{k}{|\;\varphi }_{k}=\sum_{n=1}^{N}x_{n}e^{\frac{-j2(k-1)(n-1)\pi }{N}},\;k\;=\;1,\;2,\;\cdots ,\;N\right\}$$

Let’s consider an even number for *N* first. We will interpolate **x** to generate a new sequence **z** = [*z*_1_, *z*_2_, …, *z_m_*,…, *z_M_*], where *M* > *N*. A procedure is first issued to separate the DFT sequence into two sequences of equal length, and then to pad *M*–*N* zeros in between the two sequences as shown below:

(8) $$\begin{array}{c} \phi ={\left[\phi_{1},\;\phi_{2},\;\cdots ,\;\phi_{M}\right]}^{T}\\ =\left[\underset{\begin{array}{c} \mathrm{First}\;N\;/\;2\;\mathrm{spectral}\\ \mathrm{components} \end{array}}{\underset{︸}{\varphi_{1},\;\varphi_{2},\;\cdots ,\;\varphi_{N/2}},\;}\underset{\begin{array}{c} M-N\;\\ \mathrm{zero-paddings} \end{array}}{\underset{︸}{0,\;0,\;\cdots ,\;0},\;}\underset{\begin{array}{c} \mathrm{Remaining}\;N\;/\;2\mathrm{spectral}\\ \mathrm{components} \end{array}}{\underset{︸}{\varphi_{N/2+1},\;\varphi_{N/2+2},\;\cdots ,\;\varphi_{N}}}\right] \end{array}$$

where φ denotes the zero-padded DFT spectrum. When considering an odd number of *N*, the vector φ will be constructed in a similar way, but different numbers of spectral components will alternatively be inserted, due to the uneven number of the components ahead and behind the zero-paddings:

(9) $$\begin{array}{c} \phi ={\left[\phi_{1},\;\phi_{2},\;\cdots ,\;\phi_{M}\right]}^{T}\\ =\left[\underset{\begin{array}{c} \mathrm{First}\;(N+1)\;/\;2\;\mathrm{spectral}\\ \mathrm{components} \end{array}}{\underset{︸}{\varphi_{1},\;\varphi_{2},\;\cdots ,\;\varphi_{(N+1)/2}},\;}\underset{\begin{array}{c} M-N\;\\ \mathrm{zero-paddings} \end{array}}{\underset{︸}{0,\;0,\;\cdots ,\;0},\;}\underset{\begin{array}{c} \mathrm{Remaining}\;(N-1)\;/\;2\mathrm{spectral}\\ \mathrm{components} \end{array}}{\underset{︸}{\varphi_{(N+1)/2+1},\;\varphi_{(N+1)/2+2},\;\cdots ,\;\varphi_{N}}}\right] \end{array}$$

where the first (*N* + 1)/2 spectral components are inserted ahead of the zero paddings, and the remaining (*N* − 1)/2 components are inserted behind them.

By taking the inverse discrete Fourier transformation of the frequency-domain sequence, the interpolation of its corresponding time-domain sequence can be retained with a unified sequence length:

(10) $$z_{m}\;=\;\frac{1}{M}\overset{M}{\underset{\;k\;=1}{\sum }}\varphi_{k}e^{\frac{j2(k\;-\;1)(m\;-\;1)\pi }{M}},\;m\;=\;1,\;2,\;\cdots ,\;M$$

As this is an interpolation method for upsampling a signal sequence, there are in fact numerous varieties of modified padding technique, each having different effects for dealing with the artifacts which arise due to sudden changes in the paddings [25]. As is known, these artifacts intrinsically cause problematic side effects, and they cannot be avoided. This dilemma makes the selection of a suitable alternative padding a little impracticable. Zero paddings, possessing the merits of minimal aliasing distortion, is actually the most general method for interpolation upsampling. With the upsampled sequences, the ANN postprocessor can accordingly be applied next to the preprocessor, as shown in Figure 3.

#### 2.3.2. The Convolutional Preprocessor

Gray and Goodman [26] proposed a feature-length equalizer strategy based on Fourier polynomial multiplication to maintain consistency of length of input features for artificial intelligent classifiers, as has already been reported [27]. The convolutional preprocessor will be abbreviated as follows. Recall the finite discrete convolution, in which while two sequences, **x** and **h**, are convoluted directly to result in **z** in the time domain, their corresponding frequency-domain pairs, F(x) and  F(h), are multiplied to gain F(z), i.e.,

(11) z=x∗h ⇔ F(z)=F(x)·F(h)

where **x** and **h** are two input sequences with lengths *N* and *Q* respectively. To comprehend the temporal operation, the indices of the label of the two sequences are discretized as *n*, *n* = 1, 2, …, *N*, and *q*, *q* = 1, 2, …, *Q* for **x** and **h***,* respectively. Sequence **z** corresponds to the output, indexed by *m*, *m* = 1, 2, …, *M*, of the operation. The discrete convolution of the two finite sequences is then rewritten as

(12) $$z\left[M\right]\;=\;x\left[N\right]*h\left[Q\right]\;\Leftrightarrow {\;z}_{m\;}=\;\overset{N\;+\;Q\;-\;1}{\underset{n\;=\;1}{\sum }}x_{n}h_{m\;-\;n\;+\;1}$$

The discrete convolution output *z_m_* is obtained by summing the products of the two sequences, *x* and *h*, all over the indices of *n*, where *h* has been flipped and shifted temporally ahead by *m* + 1.

The idea of the convolutional preprocessor is employed to convert the input signals to be the same in length. With the lengths *N*, *Q*, and *M* for the respective sequences **x**, **h**, and **z**, an equation can easily be set up to explain the relationship among the sequences according to (12), i.e.,

*M* = *N* + *Q* − 1 (13)

The idea of adjusting the length of output sequences can easily benefit from (12) if sequence **h** is made to be adjustable in length through the convolutional operation. While it is independent of the contents of **h**, the length of the output sequence **z** is alternatively dependent on the sequence length of **h**. For certain given *N* and *M* for sequences **x** and **z**, a nonzero sequence **h** of automatically adjustable length *M*–*N*+1 suffices to make up the difference between *M* and *N*, and makes it possible to output the stretched **z** sequences from their corresponding **x** inputs even with different lengths *N*. To preserve as much as possible the original discriminative information carried by **x**, a series of impulses, based on the operator’s intuition for the system’s impulse response, was selected for **h**. This selection could not only mitigate the loss of the original discriminative information of the input sequence but could also simplify the conversion. The impulse train of the constant sequence is, in fact, a moving average operator in the time domain or a low-pass filter in the frequency domain. Due to the variable length of **x**, the length of **h** might vary case by case. According to (13), the outputs of the preprocessor can be compensated such that they have the same expected size, a fixed length of *M*, and this can be applied accordingly as the input for the subsequent ANN postprocessor.

## 3. Materials and Methods

**Figure 4:**
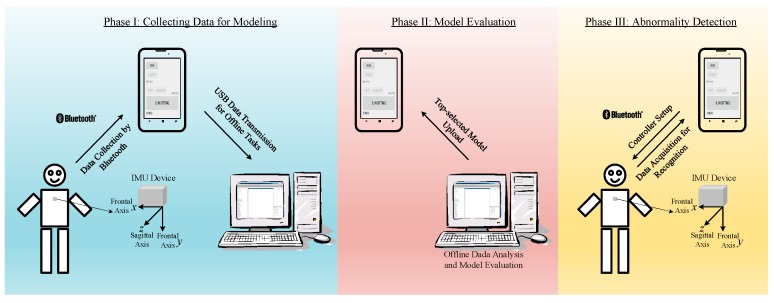
General configuration of the facilities for realistic experiments.

A Bluetooth-based IMU device was devised to record human motion for further analysis and discrimination. Wireless communication through Bluetooth benefits improved data transmission and minimizes the constraints on the volunteers’ movement. To verify and validate the developed algorithms for the abnormality detection, a desktop computer and one mobile phone were introduced, together with the IMU device, to provide the expected function. Figure 4 shows the general configuration of the devices for carrying out the experiments, as described in the following sections. The configuration in phase I was used to collect data for data analysis and model evaluation. Three kinds of model, including DTW, ANN with the DFT interpolation preprocessor, and ANN with the convolutional preprocessor, were generated based on the collected data. As offline tasks, the computational work of data analysis and model comparison was performed on the desktop computer for model evaluation. After the evaluation, an outperformed model was chosen to upload to the mobile phone, as shown in the configuration of phase II in Figure 4. With the built-in model, the mobile phone could be used alone for experiments, as shown in configuration III.

**Figure 5:**
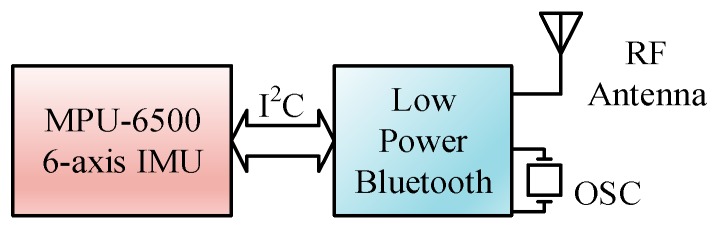
Circuit block diagram of the IMU sensor module.

**Figure 6:**
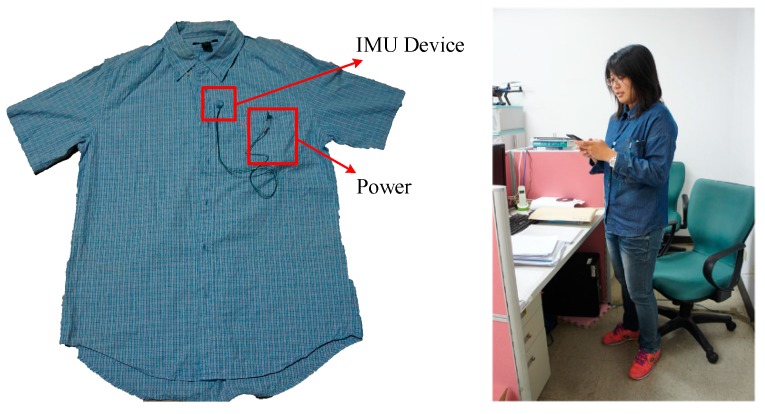
Installation of the IMU device.

The IMU device employed was designed with one sensor module, from Tjacket company [28]. The integrated device mainly includes the core sensor module and a power supply. The predominant sensor module, as shown in the circuit block diagram in Figure 5, was implemented primarily by an Invensense MPU-6500 [29], a 6-axis—3 for gyroscope and 3 for accelerator—motion tracking component. To cope with the need for real-time application, a design of the sensor module was intentionally adopted. Raw sampled data are cropped from the device at a constant 20 Hz sampling rate without any denoising process. The sparse filtering-free raw sampling scheme shows its promising tendency in terms of reducing the processing load of the IMU device, with respect to the real-time response delay, and with regard to the energy expenditure. A typical installation of the device is shown in Figure 6. A Bluetooth module was incorporated within the device for communication with the mobile phone. The hardware specifications of the mobile phone and the desktop computer are listed in Table 1.

With respect to the software modules, the remote data acquisition and MPU-6500 setup were first implemented in the mobile phone as ground primitives. As expected, the body motion of a volunteer would be primarily measured based on the acceleration in the direction of the frontal axis, which is fixed to the coordinate defined in configuration I in Figure 4. Due to the arbitrary orientation of the installed IMU device, an alignment procedure was coded into the data acquisition module as a calibration function. The calibration simply aligned the gravity direction measured upon installation with the frontal direction. It is necessary to perform a calibration before each new installation in order to achieve precise data acquisition. 

Along with the DTW direct template-matching algorithm, all the functions were coded together to form a mobile app. Using the app, the following data collection procedure was applied:

1. Confirming first the communication via Bluetooth between the IMU and the mobile phone;
2. Labeling the pattern which the volunteer wants to generate by entering a numerical code; and
3. Recording a pattern representing the motion of the volunteer by pressing the start/stop button on the app.

Six sub-functions are involved in the data collection procedure: 1. start recording, 2. motion stand-up labeling, 3. motion sit-down labeling, 4. motion walking labeling, 5. stop recording and 6. record saving and transferring. A .csv file containing the data sequences and their corresponding labels is created inside the mobile phone to accumulate the records of the test motions produced by the volunteers. The length of the acquired sequences will vary in terms of the acquired durations of each of the recorded sequences. 

**Figure 7:**
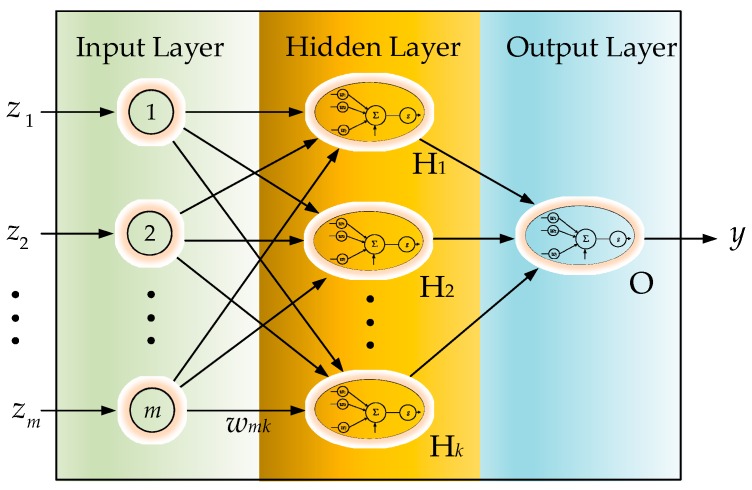
Neural network architecture.

For the validation of the ANN oriented pattern recognition methodologies, an extra training procedure would be carried out offline on the desktop computer. A three-layered BPNN with five nodes in the hidden layer, as shown in Figure 7, was adopted in this study. Using the collected datasets, training was carried out through supervised learning, developed by MATLAB and the related Toolboxes in MATLAB. A cyclic two-phase supervised learning scheme, which includes repeated training phases and validation phases, was utilized to effectively model the ANN perception. The root mean square error (RMSE), which is a common convergence criterion for adaptive cyclic learning [21], was used to evaluate the learning precision.

Although there was no extra training load, a procedure for selecting appropriate MPPs for the best template matches is still necessary in order to carry out DTW in real time. The selections of the MPPs were made as discussed in Section 4.1. 

## 4. Simulated Results and Discussions

### 4.1. Methodological Verification by Operational Elaboration

**Figure 8:**
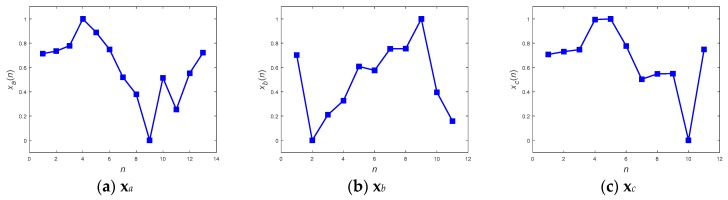
Examples in different lengths for operational elaboration.

The feasibility of the proposed methods for resolving the problem of the unequal length of the acquired patterns is examined in this section. As described in the previous sections, two approaches, including direct pattern-matching by DTW and neural network inference, have been enumerated in this study for the detection of abnormality, and two different preprocessors, including the DFT interpolation preprocessor and the convolutional preprocessor, were then designated to be situated ahead of the neural network inference in turn. Therefore, there are three different methods included in the operational illustration. Three patterns, with lengths of 13, 11, and 11, as shown in Figure 8a–c, respectively, were chosen to verify the feasibility. As their appearance shows, the patterns **x***_a_* and **x***_c_* exhibit a similar tendency, and **x***_a_* and **x***_b_* tend towards being contrary to one another.

**Figure 9:**
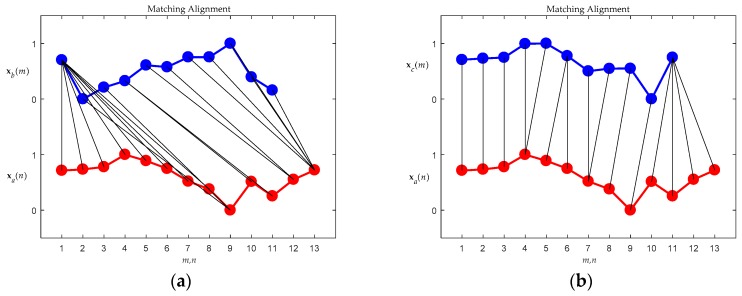
Matching alignments. (**a**) Between **x***_a_* and **x***_b_*; (**b**) Between **x***_a_* and **x***_c_*.

**Figure 10:**
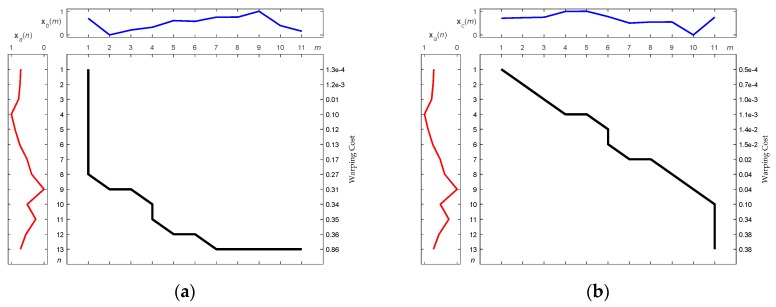
Optimal warping paths along with accumulated warping cost calculations corresponding to Figure 9. (**a**) Between **x***_a_* and **x***_b_*; (**b**) Between **x***_a_* and **x***_c_*.

Figure 9a,b illustrate the DTW match between **x***_a_* and **x***_b_*, and between **x***_a_* and **x***_c_*, respectively. There are fewer distorted alignments between **x***_a_* and **x***_c_* than between **x***_a_* and **x***_b_*, as can be seen by comparing Figure 9a,b. As shown in Figure 9, the fewer the distorted alignments, the higher the similarity between the compared patterns. This means that the motion behind **x***_c_* is closer to that behind **x***_a_* than to that behind **x***_b_*. With reference to (4) in order to calculate warping costs, Figure 10a,b show the optimal warping paths for the alignment of Figure 9a,b, respectively. As Figure 10 illustrates, a route that directly connects the upper left corner in the diagram with the lower right corner without any form of detour is the optimal match, and retains the lowest matching cost. The greater the detour the exhibited by the route, the greater the distortion implied of the point-to-point alignments (Figure 9), and the greater the expenditure of warping cost. In brief, a shorter warping path implicitly represents a lower warping cost. In the case shown in Figure 10b, it requires less warping cost than the case shown in Figure 10a. To confirm this explanation, the corresponding total warping costs for both cases were calculated as 0.86 and 0.38, respectively, i.e., the cost degrees were consistent with the eye checking of the original designated patterns.

**Figure 11:**
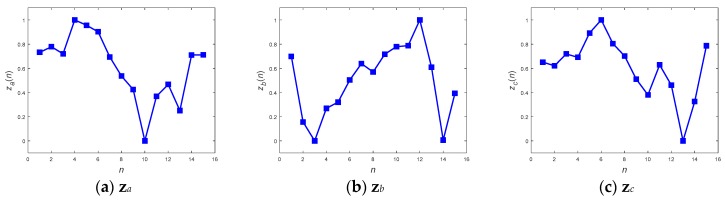
DFT interpolation preprocessed sequences **z***_a_*, **z***_b_*, and **z***_c_* from inputs **x***_a_*, **x***_b_*, and **x***_c_*, respectively.

**Figure 12:**
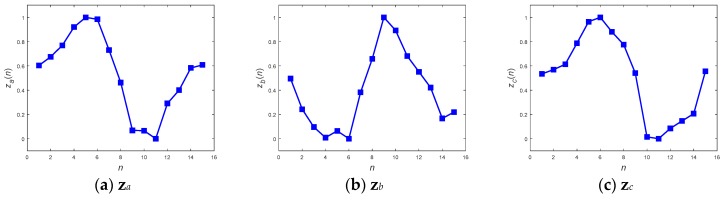
Convolutionally equalized preprocessed sequences **z***_a_*, **z***_b_*, and **z***_c_* from inputs **x***_a_*, **x***_b_*, and **x***_c_*, respectively.

With respect to the method of ANN inference, both the DFT interpolation preprocessor and the convolutional preprocessor, which were discussed in Section 2.3.1 and Section 2.3.2, were implemented first to equalize the input sequences. Figure 11 and Figure 12 present the resultant **z**s for the three inputs **x***_a_*, **x***_b_*, and **x***_c_* in Figure 8 using both of the preprocessors. Both the preprocessors ensure that the length of their output sequences will be 15 points and normalize into the range of [0, 1] for better recognition in next ANN stage. Figure 11 and Figure 12 show that all of the transformed **z**s, which have been resampled as 15-point sequences, still approximately maintain the tendency of their original inputs. This fact potentially supports confidence in the success of the corresponding pattern recognition when implementing a consecutive ANN. 

### 4.2. Recognition Performance Validations

To fairly validate the performance of the proposed methods, a *k*-fold cross-validation was carried out. For the performance evaluation, varieties of typical movements of sit-to-stand and stand-to-sit were generated by a subject, and 130 samples of these were randomly collected. As shown, the DTW algorithm only outputs values for ranking the template similarity of the testing samples. Therefore, it was accordingly necessary to implement a decision-making mechanism followed by the DTW output to label the samples into their corresponding categories. To obtain two typical sequence templates, which were regarded as the most representative delegates for the folded replicative matching, two matching pattern paradigms (MPPs) were designated from the training samples by a best-fit averaging criteria. The paradigms, labeled **z***_t_*_1_ and **z***_t_*_2_, were then used to match the unlabeled testing sample **z**_s_. The decision-making mechanism for assigning sample **z** to a specific category can thus be defined in a simple way as:

(14) $$L\left(z\right)\;=\;L\left(\underset{z_{\mathrm{ts}}}{\mathrm{argmin}}\left(\sum^{\;}\left({z,\;z}_{\mathrm{ts}}\right),s\;\in \;\left\{1,\;2\right\}\right)\right)$$

where Σ denotes the warping cost. The label function *L*(**z**) renders DTW comparable with outputs of the ANN inference. The assessment set *k* = 5 for the cross-validation, i.e., each fold contained 26 sample sequences for the circulation of the folded replications. Average accuracy was eventually produced after the five replications had been completed. Table 2 shows the performance of the folded replications and the average accuracy. 

Similar to the procedure for generating Table 2, ANN inference with both the DFT interpolation preprocessor and the convolutional preprocessor was also employed to classify the 130 acquired samples, and produced the folded reports as shown in Table 3 and Table 4. The corresponding results are found in Table 3 and Table 4 for both the DFT interpolation preprocessor and the convolutional preprocessor, respectively. Comparing the resultant accuracies across the three tables, the rate of 93.85% achieved by DTW outperformed the other two approaches. These promising results preliminarily reveal the potential of DTW in motion detection applications.

Aside from accuracy, Table 3 and Table 4 also provide the function recall execution time, taking only the recall time into account with its corresponding readily trained ANN model. In fact, the model recalling time of these two ANN approaches could be equivalent to the matching time of the DTW approach. All of these are defined as the duration required to perform the corresponding recognition function. From Table 2, Table 3 and Table 4, the five-fold average duration of 1.98 seconds achieved by DTW in Table 2 is obvious, and it was also superior to the durations (3.02 s and 3.25 s) achieved by the alternative ANN approaches, as shown in Table 3 and Table 4. Here, only 76.15 ms was required for a single DTW template matching, while 1.98 s was required for a folded matching comprising 26 samples.

### 4.3. Consideration of Model Preparation

There is one additional practical consideration when applying motion detection algorithms. As is known, for any machine learning scheme, e.g., the ANN incorporating the two preprocessors, there must be a training procedure to establish the model. With the readily trained model, a recall procedure can then be easily applied. Rather than the time to recall the model for classification, there should be an overhead training time to gain the trained model, although the training procedure is only required to be performed once for each model. Still, the minor consideration of training time has to be fairly included in the evaluation. Together with the feature preprocessing time, the ANN training time is listed in Table 5 to show the total model preparing time of both ANN models with the 130 acquired samples.

In contrast to the ANN learning approaches, DTW adopts a direct template matching scheme. No training time is required for this kind of process. Only a little model preparation time is required instead. Therefore, DTW requires a relatively shorter time for preparation. For comparison, Table 5 summarizes the total preparation time, 4.72 s, 10.66 s, and 10.72 s, for the competed models, DTW, ANN with the DFT interpolation preprocessor, and ANN with the convolutional preprocessor, respectively. Although the difference is not big in terms of the numbers’ order, DTW is still significantly more outstanding than the other two methods. In fact, the degree of significance would increase when the training samples increase. As theoretically anticipated, the learning complexity of ANN grew exponentially and led causally to a large amount of training time, even though the number of samples is only 130 in this experiment.

This analysis results in a conclusion based on the summarized data as shown in Table 5. The table reveals the excellent capability of the proposed DTW algorithm, with not only the prediction accuracy and the execution time, but also the model preparation time, which is promisingly superior to the other methods. The excellent performance of the DTW algorithm confirmed a fact that has previously been asserted: that dynamic matching provides a rapid and direct means of online activity detection.

## 5. Real-Time Application

**Figure 13:**
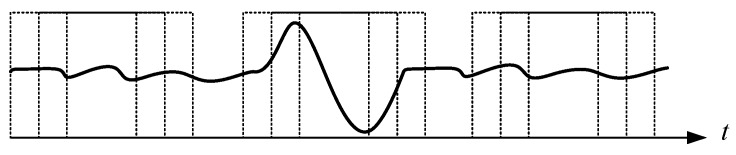
An illustration of pattern sampling and collection via the mobile app module.

As described in the previous section, the outperformed DTW was finally selected for real-time motion detection. The system was primarily devised to immediately detect three kinds of motion, stand-up, sit-down, and walking in the laboratory stage. By putting on the shirt with the installed IMU device (Figure 6), a continuous signal sequence corresponding to the motion of the volunteer was captured and passed to the mobile phone via Bluetooth. The mobile app module acted as a controller for handling the operations of the system, and also as a data acquisitor for gathering data from the IMU sensing device. Based on the sub-functions developed in Section 3, two main functions were reorganized in the mobile app for this application. The first function carried out a procedure for pattern collection and the second for real-time pattern recognition. With the main functions, the patterns could be easily discretized and collected for analysis, as shown in Figure 13.

The second function in the mobile app module performs real-time DTW pattern recognition. The selected MPPs are loaded into the mobile app, and the online detection process is then activated whenever the mobile app connects with the IMU sensing device by Bluetooth.

### 5.1. Matching Pattern Paradigm (MPP) Selection

**Figure 14:**
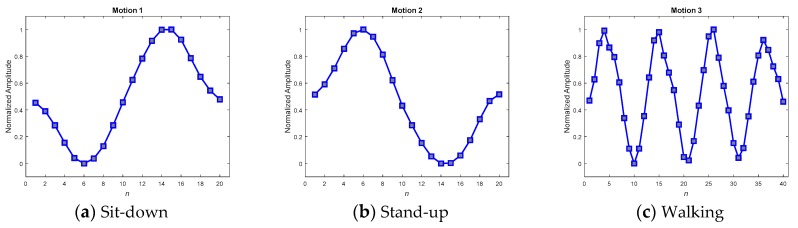
Averaged MPPs for motions 1–3, generated by the convolutional equalizer.

**Figure 15:**
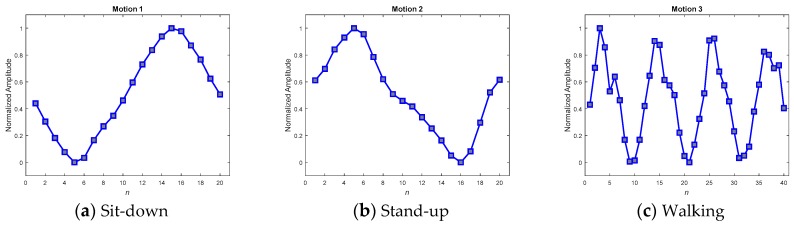
Averaged MPPs for motions 1–3, generated by the DFT interpolation equalizer.

**Figure 16:**
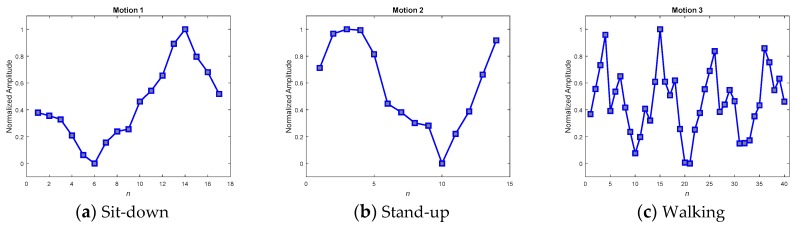
Randomly selected MPPs for motions 1–3, without any averaging procedure.

The key to achieving success in online detection is indeed the MPPs. Several methods have been developed to obtain good MPPs when testing in real scenarios. In the real scenario, ten volunteers were invited to wear the IMU sensing module to widely collect various motion patterns. Among the collected patterns, 195 samples that were admissibly representative of the three kinds of motion were then selected to average and produce the MPPs. The selection procedure comprised two steps: first, a random selection by computer; and second, a manual rejection of the relatively degraded samples in order to gain qualified representative samples. As the motion sequences of the selected samples would be of varying speed, their corresponding lengths would also differ. A DFT interpolation equalizer and a convolutional equalizer were thus employed to render each of the sequences identical in length and thus to make it possible to use them to construct a point-to-point average. Figure 14 and Figure 15 show the averaged MPPs of three kinds of motion that have been equalized by the convolution equalization and the DFT interpolation equalization, respectively. To verify the contradictory capabilities of the anti-average policy when selecting MPPs, a set of randomly selected and never-averaged patterns was chosen, as shown in Figure 16, for contrast in the experiments.

To verify the three kinds of MPP and select the best set for further application, a consensus rule was applied. This rule looked backward to support from the 195 samples, which were used to generate the MPPs, and achieved averaged recall rates for accuracies of the MPPs. Table 6 shows the averaged recall accuracies, revealing dramatically that not every averaged MPP achieved high detection accuracy. While the averaged policy, together with the DFT interpolation equalization, gained the highest accuracy of 97.69%, the one with convolutional equalization obtained only 91.54%.

Referencing these dramatic results to their corresponding MPPs in the figures, we found that the smoothest paradigms obtained by the averaged policy together with the convolutional equalization showed a downgraded matching. In contrast to the averaged policy with the convolutional equalization, the other two policies, with relatively rugged MPPs, produced relatively superior accuracy. It was realized that the averaged policy, which has relatively less modification (smoothing) of the appearance, possessed the merit of precisely detecting the motions. Hereafter, averaged candidates that were equalized by the DFT interpolation were adopted as typical MPPs for the real-time DTW abnormality detection system.

### 5.2. Performance of Real-Time Abnormality Detection

The last stumble in developing a real-time online pattern recognition system is the segmentation of the continuously captured IMU signal stream, as illustrated in the frames in Figure 13. Threshold triggers might be a good strategy for facing this problem. The mechanism sets up a set of thresholds, one for starting and the other for stopping, to commence and cease online recognition. Based on the thresholds, the function could be woken up or returned to sleep. From the viewpoint of a signal train, the duration of the function performed is an equivalent duration for segmenting a specific sequence for the purposes of matching. With this mechanism, fragmentary frames of sequences could be withdrawn from the continuous signal train, which DTW can subsequently carry out. In this study, the wake-up was assigned at a moment when the signal variation was greater than the starting threshold, and the stop assigned when the signal variation was smaller than the stop threshold lasted for at least 0.5 s. The thresholds were chosen by particle swarm optimization.

Twenty-eight volunteers, different from the ten for determining the MPPs, were invited to participate in real-time online testing. Each volunteer was requested to randomly sit down and stand up thirty times with various motion speeds. Following the completion of a series of sit-down and stand-up motions, the volunteers were requested to walk for a while. All the motions were detected and recognized automatically by the system and reported on the mobile app interface. While the sit-down and stand-up motions represent exceptional subjects, the walking crudely stands for an ordinary normal motion. Observations of the experiment showed that most walking tests could be matched in general. For statistics, only the predictions of sit-down and stand-up were taken into account for the determination of accuracy. 

Table 7 shows the real-time test results. The average accuracy of 88.63% for both sit-down and stand-up indicates that the DTW algorithm shows significant potential with respect to online motion detection applications. A real-time level of motion detection was also shown by considering the template matching time 76.15 ms, as approximated in Section 4.2. It took only an insignificant 5.68% from the average response time of 1.34 s achieved by the mobile app.

## 6. Conclusions

The DTW technique for motion detection has been greatly developed, as reported in this study. Based on the experimental results in this study, one can transparently realize the merits of DTW in contrast and comparison with the other two alternative ANN pattern recognition approaches. This study clarified that a direct template-matching method could be superior to any other learning scheme for online detection of body motions using an IMU device in addition to DTW, which is often regarded as the best direct template-matching technique. The superiority of DTW could make it possible to develop applications such as the detection of abnormality incorporating the IMU device. Thus, based on the present study, it is expected that an Internet of Things (IoT)-based assistive device could be successfully used for remote medical care.

## Figures and Tables

**Table 1:** Specifications of the mobile phone and the desktop computer.

Specifications of Mobile Phone		Specifications of Desktop Computer	
Model	ASUS ZenFone ZC500TG	Processor	Intel i7-3770, 3.40 GHz
Processor	MediaTek 6580 QuadCore, 1.3 GHz	Memory	DDR3-1600 8 GB
Memory	2GB RAM	Storage	Toshiba SATAII 1TB, 7200 rpm
Storage	8GB eMMC Flash	Operation System	Microsoft Windows 7
Operation System	Android 5.1	Display	AMD Radeon HD 6670, 2 GB
Display	1280 × 720 pixels, 5 inches	USB	USB 3.0

**Table 2:** *k*-fold cross-validation for DTW approach.

Training Folder	Validation Folder	Correct Count	Error Count	Execution Time (sec)	Accuracy (%)
#1, #2, #3, #4	#5	24	2	1.84	92.31
#1, #2, #3, #5	#4	25	1	1.95	96.15
#1, #2, #4, #5	#3	24	2	1.76	92.31
#1, #3, #4, #5	#2	25	1	2.21	96.15
#2, #3, #4, #5	#1	24	2	2.13	92.31
**Average**		24.4	1.6	1.98	93.85

**Table 3:** *k*-fold cross-validation for the approach of ANN with the DFT interpolation preprocessor.

Training Folder	Validation Folder	Correct Count	Error Count	Execution Time (sec)	Accuracy (%)
#1, #2, #3, #4	#5	22	4	3.58	84.62
#1, #2, #3, #5	#4	24	2	2.84	92.31
#1, #2, #4, #5	#3	22	4	2.93	84.62
#1, #3, #4, #5	#2	23	3	2.74	88.46
#2, #3, #4, #5	#1	24	2	3.37	92.31
**Average**		22.75	3.25	3.02	87.50

**Table 4:** *k*-fold cross-validation for the approach of ANN with the convolutional preprocessor.

Training Folder	Validation Folder	Correct Count	Error Count	Execution Time (sec)	Accuracy (%)
#1, #2, #3, #4	#5	22	4	2.74	84.62
#1, #2, #3, #5	#4	24	2	3.53	92.31
#1, #2, #4, #5	#3	21	5	3.48	80.77
#1, #3, #4, #5	#2	23	3	2.92	88.46
#2, #3, #4, #5	#1	23	3	3.56	88.46
**Average**		22.6	3.4	3.25	86.92

**Table 5:** Performance conclusion in both accuracy and execution time.

Model	Prediction Accuracy (%)	Prediction Execution Time (sec)	Model Preparing Time (sec)		
			Feature Preprocessing Time (sec)	ANN Training Time (sec)	Total Model Preparing Time (sec)
DTW	93.85	1.98	−	−	4.72
ANN with Preprocessor 1 *	87.5	3.02	3.6	7.06	10.66
ANN with Preprocessor 2 **	86.92	3.25	1	9.72	10.72

* Preprocessor 1: DFT interpolation preprocessor; ** Preprocessor 2: convolutional preprocessor.

**Table 6:** Comparison of matching pattern paradigm selection methods.

Method to Generate Matching Pattern Paradigm	Accuracy (%)
Averaging with Convolutional Equalizer	91.54
Averaging with DFT Interpolation Equalizer	97.69
Randomly Selection without Averaging	95.38

**Table 7:** Performance of the online real-time pattern recognition.

Motion	Test Count of Every Volunteer	Averaged Missed Count	Averaged Error Count	Averaged Accuracy (%)	Averaged Time to Response (s)
Sit-down	30	2.79	0.86	87.86	1.17
Stand-up	30	2.07	1.11	89.40	1.52
Average	30	2.43	0.98	88.63	1.34
